# The two faces of elderspeak

**DOI:** 10.1038/s44400-026-00072-0

**Published:** 2026-07-15

**Authors:** Adolfo M. García

**Affiliations:** 1 Centro de Neurociencias Cognitivas, Universidad de San Andrés, Buenos Aires, Argentina; 2 Global Brain Health Institute (GBHI), University of California, San Francisco, San Francisco, CA, USA and Trinity College Dublin, Dublin, Ireland; 3 Departamento de Lingüística y Literatura, Facultad de Humanidades, Universidad de Santiago de Chile, Santiago, Chile

**Keywords:** Health humanities, Psychology, Psychology

## Abstract

Elderspeak is a collection of prosodic, lexical, and syntactic adaptations adopted for communicating with older adults. Often rejected as ageist and patronizing, it actually has mixed effects: some features hinder comprehension and well-being, while others aid understanding, social connection, or care. Considering seniors with and without dementia, this perspective addresses motivations, forms, and impacts of elderspeak, advancing a personalized, science-first agenda to preserve benefits despite flaws.

## Introduction

Like other cognitive domains, language changes noticeably in the course of aging and dementia^1^. For instance, healthy elders know more words than younger adults^2^, but they are slower to process them^3–5^ and they struggle to grasp fast-paced or noise-laden utterances^6^. Word access delays are exacerbated in persons with Alzheimer’s dementia, which further compromises vocabulary knowledge and semantic memory at large^7^. In both groups, these patterns are accompanied by pragmatic and discourse-level challenges^1^, delineating particular idiolects across individuals.

Recognition of such declines, alongside stereotypes of older age, may lead interlocutors to adopt elderspeak^8^, a speech register aimed to enhance senior-directed communication. Nevertheless, the impact of elderspeak is far from consistent, with prominent views emphasizing its negative aspects despite evidence of positive effects^8^. In this perspective, I consider key features, motivations, and impacts of elderspeak, calling for a nuanced, person-centered approach to the construct.

## The nature and impact of elderspeak

First reported in nursing homes^9,10^, elderspeak is typified by partly predictable linguistic adaptations^8^. Speech is delivered more slowly amidst longer pauses. Pitch becomes higher and intonation is exaggerated. Short, frequent words are favored over long, uncommon ones. Sentences are shortened and syntax is simplified, often with repetitions, tag questions, and minimizers (e.g., *just*). Specific forms of address prove unusually recurrent, including terms of endearment (e.g., *sweetie*) or the substitution of first-person plural pronouns (*we*) for second-person singular forms (*you*)—a phenomenon known as collective framing.

Typically, instances of elderspeak are typified by the co-occurrence of many such features within complete utterances. For example, a caregiver assisting with dressing may say “*Okay sweetheart, we’re going to put our sweater on now, aren’t we? Let’s lift our arm—good job!*” In a different care context, such as medication administration, a nurse might remark “*Alright dear, here comes our pill. We take it nice and easy, okay?*” These adjustments occur widely in health care settings^11–13^, appearing in almost one every four utterances delivered to seniors^10^ and in two out of three nursing home conversations^14^. Dialog between older adults and professional caregivers can vary in their degree of reliance on elderspeak, as shown in Table 1, which reproduces real-life exchanges at UK hospital wards.

**Table 1:** Real-life interactions between older adults with dementia and caregivers, ranked by reliance on elderspeak (per Bridgstock, 2018)^54^

Example	Elderspeak elements	Reliance on elderspeak
PLWD: How d’you do this? You do it. CG: Say again, darling. PLWD: how d’you do? Checking myself. CG: Just check the chest. It’s not infected or anything. PLWD: Ah.	1. Term of endearment	Light
CG: We just have to use the sliding sheet, just to slide you up the bed. CG: Is that all right? PLWD: Okay	1. Collective framing 2. Minimizer 3. Minimizer	Moderate
CG: Come on, lovely? CG: Just relax. PLWD: You’re fucking hurting me. CG: We’re nearly finished now. CG: Okay?	1. Term of endearment 2. Minimizer 3. Collective framing 4. Tag question	Heavy

Speaker codes in the original transcripts were replaced with ‘person living with dementia’ (PLWD) and ‘caregiver’ (CG) for clarity. Punctuation was modified to accommodate it to standard conventions.

Per some accounts^15^, elderspeak is triggered when speakers notice physical cues (e.g., hair color, wrinkles) or communicative features (e.g., voice quality, word choice) indicative of old age. This register is particularly pervasive when older adults are perceived as functionally or cognitively compromised^13,16,17^. It is more frequently adopted by younger than by older adults^18,19^, though it can be observed even in children^20^. Through it, caregivers aim to foster comfort, warmth, cooperation, or comprehension^21^. Yet, these goals are, at best, inconsistently met.

In several studies, older adults have described elderspeak as patronizing, disrespectful, and controlling^22–25^. Early reports, indeed, found that most instances of elderspeak were indistinguishable from motherese—i.e., infant-directed speech^8^. For some elders, specific adaptations, such as exaggerated prosody, diminish comprehension^26^ and increase physiological stress responses^27^. The impact of elderspeak is most profound in persons with dementia, who may be driven to silence, tears or withdrawal^28,29^. In fact, elderspeak has been found to double the likelihood of resistiveness to care in this group^13^. On account of these findings, the construct has often been deemed a disruptive, ageist practice.

Notwithstanding, some components of elderspeak seem to favor communication. Wayfinding and instruction-following can be facilitated by reducing sentence length and complexity^19,30^. Comprehension may also be boosted via semantic elaborations, including repetition and extended explanations^26^. Further communicative benefits have been found through strategic use of focal stress and slowed speech rate^31,32^. In specific studies, exaggerated prosody was actually deemed supportive by senior addressees^33^, and elderspeak, in general, was perceived to promote social bonds between older adults and caregivers^34^. Accordingly, a full-fledged rejection of elderspeak seems unwarranted.

## A balanced view, an actionable agenda

Overall, while elderspeak, at large, is considered an ageist, infantilizing practice^1^, some of its components should be given strategic consideration. Yet, the task is not to enumerate features that are universally negative or positive. Indeed, as seen above, one and the same adjustment (e.g., exaggerated prosody) may have positive or negative consequences depending on the context, the interlocutors, and, no less importantly, the very interaction of linguistic choices. Syntactic simplification, for instance, can foster comprehension independently of negative social evaluation, whereas exaggerated prosody disproportionately contributes to perceptions of infantilization, particularly when bundled with other accommodative features^26,35,36^. Even structurally complex utterances may be perceived as condescending when paired with exaggerated intonation^32^. Likewise, reductions in clause density can facilitate processing in the absence of prosodic exaggeration^26^. Accordingly, patronization appears to emerge from the *co-occurrence* of cues that collectively frame the interaction as over-accommodative rather than from any isolated linguistic feature.

Psycholinguistic insights illuminate these communicative dynamics. Perceptions of accommodation are likely driven by cue-integration processes in which listeners jointly evaluate structural complexity and paralinguistic modulation to infer speaker intent and interlocutor status^37,38^. Syntactic simplification primarily reduces processing load, whereas prosodic modulation operates as a higher-level interpretive cue that shapes how structural adjustments are encoded and evaluated during comprehension^39,40^. When prosodic signaling remains neutral, reductions in syntactic complexity are likely to be processed as task-oriented support. Conversely, when marked prosodic exaggeration is present, the same structural features are reinterpreted as reflecting diminished competence rather than transient processing needs, consistent with models in which prosody constrains pragmatic inference and social meaning^41,42^. Under this view, the boundary between supportive accommodation and patronizing over-accommodation arises when prosodic cues dominate cue-weighting processes, shifting interpretation from facilitative processing support to a face-threatening attribution of global incapacity^15,43^.

Granted, these processes do not manifest identically across individuals. Tactful probing is required to identify whether, for a given older adult, a specific linguistic adaptation proves pernicious or helpful. Importantly, evidence of negative effects of elderspeak does not mean that senior-sensitive verbal adaptations should be abandoned. In fact, neglect of this group’s socio-interactive needs can erode communication and health outcomes^44,45^. Hence, just as diagnostics and therapeutics call for personalized frameworks, so too does elderspeak call for approaches centered on individual preferences and traits.

**Fig. 1:**
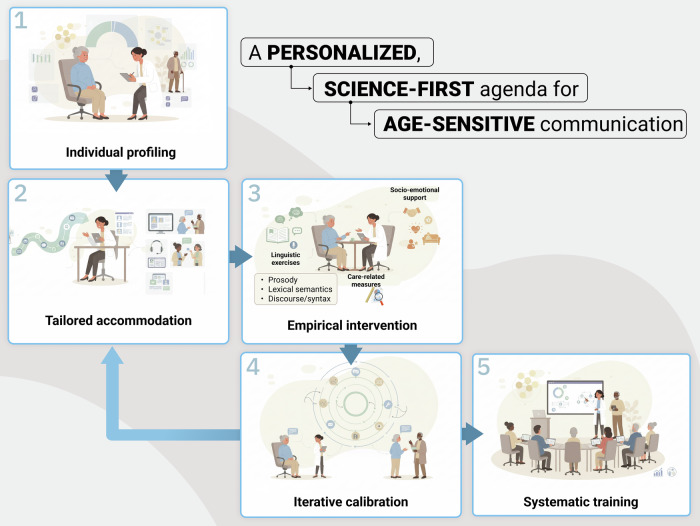
Conceptual workflow for a personalized, evidence-driven approach to age-sensitive communication. The model progresses from comprehensive profiling of older adults’ communicative capacities to the design of individualized, theory-informed accommodations. These accommodations are tested through empirically grounded interventions spanning linguistic, socio-emotional, and care-related domains. Measurable outcomes span interactional, behavioral, and emotional domains, including key prosodic, lexico-semantic, syntactic, and discursive dimensions. Observed outcomes inform iterative refinement of communication strategies, which are ultimately consolidated into scalable training programs for caregivers and professionals.

Moving forward, a personalized, science-first agenda can be advanced to promote effective age-sensitive communication. Core actions would be organized into five components, namely: individual profiling, tailored accommodation, empirical intervention, iterative calibration, and systematic training (Fig. 1).

First, individual profiling is needed due to the vast heterogeneity of the older adult population. Rather than assuming uniform communicative decline, this component emphasizes systematic assessment of each individual’s sensory, cognitive, linguistic, and neurological profile. The motivation lies in avoiding stereotype-driven overaccommodation by grounding communication strategies in measurable capacities, such as hearing acuity, processing speed, lexical access, syntactic comprehension, and dementia status. A multidimensional communicative profile can be established through neuropsychological, psycholinguistic, and clinical linguistic assessments. By establishing an evidence-based baseline, the model ensures that subsequent adaptations respond to actual needs rather than perceived age-related deficits, laying the foundation for personalized and respectful age-sensitive communication.

Second, personalized communicative profiles should inform tailored accommodations. This component entails mapping individual strengths and limitations onto specific adjustments across prosodic, lexical, syntactic, and discourse domains. For example, slowed speech rate may be prioritized for individuals with preserved vocabulary but delayed processing, whereas lexical simplification may be emphasized in dementia with semantic decline. The panel underscores theory-informed decision-making, ensuring that accommodations are selective rather than bundled indiscriminately. Its motivation is to prevent maladaptive cue combinations—such as pairing syntactic simplification with exaggerated prosody—by designing coherent, need-driven strategies.

Such strategies should be captured in testable hypotheses, initially derived from the existing literature. Elderspeak-related outcomes should depend on cue combinations rather than isolated features: syntactic simplification (e.g., reduced clause density) should improve comprehension and task compliance only when prosody remains neutral, whereas the same structural simplifications will increase perceptions of patronization and resistiveness to care when paired with exaggerated intonation and infantilizing address forms. Also, prosodic exaggeration should exert a disproportionate effect on social evaluation relative to comprehension, such that even linguistically complex utterances will be judged as condescending when delivered with marked pitch elevation and affective modulation. In addition, individual cognitive profiles should moderate intervention efficacy. For example, the combination of delayed word access^3–5^ and increased word knowledge^2^ in healthy elders suggests that, for this group, slower production may be more adaptive than simplified choices. Conversely, parallel decays in word access speed and word knowledge^1^ in persons with dementia arguably calls for both slower utterances and easily retrievable words.

Third, such hypotheses should be implemented as empirical interventions. This component entails deploying customized communication strategies in naturalistic care or conversational settings and systematically documenting their use. Distinct maneuvers should be established across linguistic and socio-emotional domains, including prosodic modulation, address forms, sentence structure, and explanatory style. Naturalistic interactions should be recorded, analyzed, and coded –ideally, with specialized tools, like the Toolkit to Examine Lifelike Language (TELL, an app that quantifies multiple speech and language features)^46,47^ and the Iowa Coding Scheme for Elderspeak (ICSE, a framework for tagging multiple aspects of this register)^48^. Key target dimensions, available through TELL and the ICSE, include (i) prosodic markers (e.g., speech rate, pause frequency and duration, pitch range, intensity variability), (ii) lexico-semantic measures (e.g., lexical diversity, word frequency, semantic specificity), and (iii) discourse-level indices (e.g., utterance length, syntactic complexity).

Fourth, ensuing outcomes should give rise to iterative calibration. Focused feedback sessions, led by a communication expert, should then be held for caregivers to see their speech and language metrics alongside other relevant measures. Profiles showing excessively slow speech, dense pausing, and markedly reduced lexical diversity may indicate over-accommodation, prompting coaching toward more natural prosody or richer lexical choices. Conversely, evidence of rapid delivery, long syntactic units, or high lexical specificity in the context of comprehension difficulties may motivate selective slowing or simplification. Over repeated interactions, changes in these objective measures can be interpreted alongside behavioral and emotional outcomes (e.g., engagement, agitation, cooperation) to iteratively refine communicative strategies. When needed, resulting adjustments should be framed as new testable hypotheses, leading back to the second component.

Finally, the most successful practices should be integrated into scalable educational programs for care providers. Deployment begins by formalizing empirically validated communicative practices into structured guidelines, training manuals, decision trees, and digital resources tailored to specific care contexts. These materials should translate abstract principles into actionable recommendations, such as when to slow speech versus simplify syntax, or how to modulate prosody without triggering patronization. Crucially, such resources must be conceived as dynamic, updatable documents rather than static protocols. As new evidence emerges, populations change, or communicative demands evolve across stages of aging and dementia, guidelines should be periodically revised, versioned, and expanded. Embedding feedback mechanisms—drawing on outcome metrics, caregiver reports, and patient responses—ensures continuous refinement. In this way, scalation preserves the model’s science-first, person-centered ethos while enabling sustainable, system-wide impact across institutions and professional training programs.

As stakeholders navigate this framework, healthy and pathological aging must be recognized as dynamic phenomena. Consider the case of people living with dementia, whose linguistic^1^, cognitive^49^, and socio-emotional^50,51^ capacities change from mild to severe stages. In early-stage dementia, when pragmatic awareness and social self-concept are relatively preserved, exaggerated prosody or collective pronouns may be perceived as patronizing and autonomy-threatening, even if structural simplification offers processing support. As dementia advances and metalinguistic awareness declines, however, the same prosodic and directive cues may become functionally necessary to scaffold attention, regulate affect, or initiate action, with reduced sensitivity to their social implications. Conversely, accommodative strategies that are supportive in severe dementia—such as highly directive phrasing or repeated prompts—may be unnecessary or counterproductive earlier in the disease course. This dynamic perspective underscores that effective age-sensitive communication is not only person-centered but also stage-aware, requiring periodic reassessment and recalibration as dementia progresses.

Importantly, existing intervention studies attest to the potential of coaching caregivers in age-sensitive communication. For example, in the training program by Shaw, Williams, and Perkhounkova^52^, nursing home staff received structured education on identifying concrete elderspeak features. These included collective pronouns (e.g., “*we need to take our medicine*”), exaggerated intonation, diminutives, and directive tagging. They were then trained to replace them with adult-directed alternatives, including neutral prosody, direct second-person address, and task-focused explanations delivered at a slower but non-infantilizing rate. Training involved didactic sessions, video-based exemplars contrasting elderspeak with respectful accommodation, and guided practice with feedback. By reducing patronizing cues that trigger agitation and refusal, staff were able to complete care tasks with less behavioral disruption while further decreasing reliance on antipsychotic medication. Ideally, further works in this line would help define thresholds between effective and maladaptive communicative adjustments, acknowledging sociocultural and language-specific constraints^53^ alongside individual needs and styles.

## Conclusion

In sum, language skills change markedly throughout aging and dementia, prompting communicative adaptations from interlocutors. Some such adjustments reflect stereotypes of old age, while others represent cognitively sensible intuitions. Optimal strategies can only be reached by acknowledging the interlocutor’s personal skills, predilections, and traits, especially upon considering basic science insights. A personalized, science-first ethos can ensure that elderspeak does not fall short of its good intentions and that its benefits are not masked by its imperfections.

## Data Availability

No datasets were generated or analyzed during the current study.
